# An in-depth analysis of antimicrobial prescription quality in 10 non-university hospitals, in southwest Germany, 2021

**DOI:** 10.2807/1560-7917.ES.2024.29.46.2400156

**Published:** 2024-11-14

**Authors:** Gesche Först, Roland Giesen, Geertje Fink, Matthias Sehlbrede, Nicole Wimmesberger, Rebekka Allen, Kerstin Meyer, Sabine Müller, Hanna Niese, Sina Polk, Barbara Reistle, Carolin Schuhmacher, Andreas von Ameln-Meyerhofer, Kim Winter, Dave Wirth, Winfried V Kern, Erik Farin-Glattacker, Siegbert Rieg, the ID ROLL OUT Study group, Other members of the ID ROLLOUT study group, Stephan Horn, Freba Khaleqi, Miriam Kiefer, Matthias Löffler, Susanne Mertins, Michael Schmid, Arno Schmidt, Anna-Teresa Tremmel, Florian Wagner, Christian van Uden, Ulrike Witten-Stephan, Yvonne Wuwer

**Affiliations:** 1 Division of Infectious Diseases, Department of Medicine II, Medical Center – University of Freiburg, Faculty of Medicine, University of Freiburg, Freiburg, Germany; 2 Clinical Pharmacy, Institute of Pharmaceutical Sciences, University of Freiburg, Freiburg, Germany; 3 Section of Health Care Research and Rehabilitation Research, Faculty of Medicine and Medical Centre, University of Freiburg, Freiburg im Breisgau, Germany; 4 Pharmacy Service, Hospitals Ostalb, Mutlangen, Germany; 5 Department of Pharmacy, Hegau-Bodensee-Hospital Singen, health association Landkreis Konstanz, Germany; 6 Pharmacy Service, St. Josefshospital, Freiburg, Germany; 7 Pharmacy Service, Alb-Fils-Hospitals, Göppingen, Germany; 8 Pharmacy Service, Marienhospital Stuttgart, Stuttgart, Germany; 9 Pharmacy Service, Schwarzwald-Baar hospital, Villingen-Schwenningen, Germany; 10 Pharmacy Service, clinic group southwest, Sindelfingen, Germany; 11 Pharmacy Service, clinic group southwest, Böblingen, Germany; 12 Pharmacy Service, Hospital Mittelbaden, Rastatt-Forbach, Germany; 13 The members of the ID ROLL OUT Study group are listed under Collaborators

**Keywords:** point prevalence survey, antibiotic prescribing, antimicrobial stewardship, antibiotic stewardship, quality indicators, AWaRe classification

## Abstract

**Background:**

Non-university hospitals are the major provider of inpatient care in Germany, serving 89% of acute care hospital beds. Although surveillance data on antimicrobial use in hospitals are widely available, data on prescription quality are rare.

**Aim:**

We aimed to provide an in-depth analysis of antimicrobial prescribing patterns and quality in southwest German non-university hospitals.

**Methods:**

During 2021, we performed three point prevalence surveys (PPS) in 10 non-university hospitals, representing ca 10% of hospital beds in the federal state of Baden-Württemberg (11 million inhabitants). Demographic and clinical information were collected. We assessed the overall performance of 14 validated process quality indicators (QI) covering infection diagnostics, antimicrobial therapy and documentation.

**Results:**

Of 8,560 patients analysed, 2,861 (33%) received at least one antimicrobial. Most (2,789, 80%) antimicrobial prescriptions were for therapeutic indications. Most frequently prescribed agents were beta-lactam/beta-lactamase inhibitors (1,120, 40%) in therapeutic and cefuroxime (269, 37%) in prophylactic indications. According to the World Health Organization’s Access, Watch, Reserve classification, the Access-to-Watch ratio was 0.73. Overall adherence to QIs was low and varied substantially (27–93%), with documentation, possible streamlining and switching to oral therapy exhibiting the lowest fulfilment rates (< 50%).

**Conclusion:**

The results indicate a need to improve antimicrobial prescribing quality in non-university hospitals. The high prevalence of antimicrobial use in our setting underlines the demand for sustainable antimicrobial stewardship programmes in this sector. Our QI-based PPS approach can be used to identify key targets for future antimicrobial stewardship interventions. The results indicate a need for further legislation on antimicrobial stewardship.

**Key public health message**

**What did you want to address in this study and why?**

Studies show that three of 10 hospital patients receive antimicrobial (AM) prescriptions and between 30 and 50% of treatments are inappropriate. Non-university hospitals provide 90% of inpatient care in Germany, but there is little data on their use of AMs. We therefore investigated AM prescribing quality to identify potential key areas for improvement in non-university hospitals in our region.

**What have we learnt from this study?**

Our results indicate that the quality of AM prescribing in non-university hospitals needs to be improved for the majority of therapies. Based on our findings, special attention should be paid to timely, documented treatment planning in the future. Compared with some other Western European countries, the choice of AMs should be optimised in favour of the use of AM with a narrower spectrum of activity.

**What are the implications of your findings for public health?**

The study confirms that the quality of AM prescribing in hospitals should be monitored regularly, as recommended by the national guidelines. The method we used may serve as a valuable tool to identify key targets for future interventions. Our findings emphasise the need for sustained antimicrobial stewardship programs and implementation of infectious diseases specialist services also in non-university hospitals in southwest Germany.

## Introduction

The global burden of disease from the progressive spread of bacterial antimicrobial resistance (AMR) is substantial [1] and the overuse of antimicrobials (AM) leads to a further increase in AMR [2]. In addition to the impact on AMR rates, inappropriate AM use is associated with increased morbidity and mortality [3]. Furthermore, it has been shown that 30–50% of AM therapies are inappropriate [4].

Antimicrobial stewardship (AMS) programmes are designed to ensure high-quality AM prescribing at patient level and thereby counteract the selection of AMR. Prior quality analysis of AM prescriptions is an elementary step in the planning of AMS interventions [5]. Point prevalence surveys (PPS) have proven to be a valuable tool for assessing the appropriateness of AM prescriptions [6]. International PPS are carried out regularly in a large number of hospitals around the world (global PPS [7]) and specifically in Europe (European Centre for Disease Prevention and Control (ECDC) PPS [8]). These PPS measure both the prevalence of AM use and the prevalence of hospital-acquired infections, as well as address certain questions about the appropriateness of prescribing. Repeated PPS help to identify prescribing trends and thus evaluate the effectiveness of AMS interventions [5]. Unfortunately, definitions of the appropriate use of AM vary widely in the literature [9]. Often, adherence to local guidelines is used as a benchmark for appropriate therapy [9]. However, the sole fulfilment of a binary category - guideline-compliant therapy or not - does not reflect the multiple quality levels of a drug prescription. For this reason, the use of quality indicators (QI) to more accurately assess the appropriate use of AM has been proposed [5,6,9,10].

Despite a comprehensive national guideline and a popular AMS education initiative, AMS programmes in Germany are established to varying and often limited extents [11]. Infectious disease (ID) specialists, who often promote and lead AMS teams are also rarely available, especially in non-university hospitals [12,13]. Moreover, little is known about the quality and appropriateness of AM therapies in German hospitals [14,15]. Hitherto, studies have been predominantly conducted in large, academic and tertiary care institutions (often after, or alongside, the implementation of AMS measures) rather than in non-university hospitals [16-18].

As in most European countries, non-university hospitals account for the vast majority of hospital beds (in Germany 89% of acute care beds) and, as a consequence, for the vast majority of AM prescriptions [19]. This study therefore aims to investigate the prevalence and quality of AM prescribing patterns in a sample of 10 non-university hospitals in southwest Germany.

## Methods

### Study design and setting

The study was conducted within the framework of the ID ROLL OUT study - a multi-centre interventional study on the implementation of AMS programmes and ID specialist services [20]. We included all secondary or tertiary acute care hospitals in southwest Germany (n = 10) participating in the ID ROLL OUT study. These were all non-university hospitals and had no comprehensive structured prior AMS activities. Their baseline AMS activity was quantified using a German adaptation of the ICATB2 score (a composite score for AMS framework, resources and action) [21] and resulted in nine hospitals with the classification ‘E: very poor’ and one hospital with the classification ‘D: poor’ (AMS-GER Score according to Giesen et al. [22]). Moreover, these hospitals did not offer standardised ID specialist services.

We developed a detailed PPS to determine the appropriateness of AM therapies overall, and also accordance with individual relevant QIs [15,23-26].The PPS was conducted three times at each participating hospital - once during the second quarter (Q2: April–June), once during the third quarter (Q3: July–September) and once during the fourth quarter (Q4: October–December) of 2021. The participating hospitals represent ca 10% of all hospital beds in the federal state of Baden-Württemberg (population 11.1 million). They were distributed geographically over the federal state, including rural and urban settings. The hospitals varied in size and structure, with the number of beds in each hospital ranging from 260 to 835, with a median of 401. Further hospital characteristics are given in Supplementary Table S1.

### Data collection

Data were collected at each hospital by a locally composed interdisciplinary team consisting of physicians skilled in AMS and hospital pharmacists. Prior to the start of the study, the team who would be carrying out the survey completed the advanced Antimicrobial Stewardship training courses initiated by the German Society for Infectious Diseases. The interdisciplinary teams were trained on using the PPS by senior AMS clinical pharmacists at the University Medical Center Freiburg (UniMedCentFR) via specific education scenarios. A user manual was provided and a trial period for data entry was offered before the beginning of the data collection.

During the survey period, the surveyors had the possibility to contact senior AMS clinical pharmacists and ID specialists at the UniMedCentFR at any time, if further consultation was needed.

The survey was based on the framework of the global PPS project [7]. At 08:00 on the day of the survey, all adult inpatients (> 18 years), were screened for a prescription of at least one systemic AM (antibiotic or antifungal) and for surgical antibiotic prophylaxis within the previous 24 hours. For each patient, demographic data, information on the prescribed AM related to the underlying infection, diagnostic management, treatment and documentation were taken from their medical records. Information that was not yet available on the day of the survey (e.g. duration of therapy) was collected after the patient’s case was closed. The data were entered pseudonymised into an online datasheet using REDCap (Research Electronic Data Capture) tools hosted at the UniMedCentFR [27]. All hospital wards were assessed with the exception of psychiatry, rehabilitation and other long-term care wards. Detailed information about the wards and departments is given in Supplementary Table S2.

### Data evaluation

A senior AMS clinical pharmacist from the UniMedCentFR carried out a plausibility check of the dataset. Data collected from the surveys in Q2, Q3 and Q4 of 2021 were aggregated and analysed in this aggregated form. Demographic details, prevalence of AM use, choice of antimicrobial substances, route of administration, indication for antimicrobial prescribing and infections were summarised descriptively. Demographic details and the prevalence of AM use were reported at patient level. The prevalence of AM use was defined as the number of patients receiving antimicrobial drugs at the time of the survey divided by the total number of surveyed patients and expressed as a percentage. Other information was reported at prescription level. Prevalence of AM prescribing was summarised at department level and by ward type. The AM use prevalence per ward type is given as median prevalence of all 10 hospitals. This avoids distorting the results in favour of hospitals with a particularly large number of patients.

In this study, we used the World Health Organization (WHO) AWaRe (Access, Watch, Reserve) classification to describe prescribing patterns and to analyse differences in the prescribing quality between AM groups [28].

### Quality of antimicrobial prescribing

Using the collected PPS data, we measured the fulfilment of 14 process QIs. The QIs were selected because they were either validated in the literature [29] and/or had been recommended by the German AMS guidelines [5]. Table 1 gives an overview of the QI definitions. The selected QIs were additionally validated within the study setting (see inter-rater reliability).

**Table 1:** Process quality indicators used to quantify antimicrobial prescribing quality in 10 non-university hospitals, southwest Germany, 2021

Category quality indicator	Data evaluation details
Diagnostics	
Adequate blood culture diagnostic before therapy [24,38,41-43]	Per drug evaluated on day of the survey
Adequate microbiological diagnostic [38,42-46]	Per drug evaluated on day of the survey
Therapy	
Indication confirmed [38,41]	Per drug evaluated on day of the survey
Infection confirmed/ highly probable [41,44]	Per drug evaluated on day of the survey
Adequate dose with regard to the infection [45]	Per drug evaluated on day of the survey
Dose adjustment to renal function [24,38,41,46]	Per drug evaluated on day of the survey
Appropriate drug choice [24,38,41,42,46]	Per patient evaluated on day of the survey
No further streamlining possible [24,38,41,42,46]	Per patient evaluated on day of the survey
Adequate duration of AM therapy (until day of the survey) [38,41,45-47]	Per patient evaluated on day of the survey
Duration of perioperative prophylaxis (maximum 24 hours) [41,44,45]	Per drug evaluated retrospective
No oral switch possible (for parenteral therapy with an AM with high oral bioavailability) [24,38,41-43,46]	Per drug evaluated on day of the survey
Documentation within 72 hours	
Planned treatment duration [24,42,45]	Per drug evaluated retrospective
Site of infection [24,42,45]	Per drug evaluated retrospective
Treatment reevaluation [45,48]	Per patient evaluated retrospective

AM: antimicrobial.

Therapies for which certain QIs were not applicable, or where data were missing/unknown, were excluded from the respective analysis. More details on the calculation algorithm of the QIs are provided in the Supplementary Table S3.

Appropriateness of dose, drug choice, duration of AM therapy and microbiological diagnostics were evaluated according to local guidelines. The guidelines have been developed by experts at the UniMedCentFR based on current national and international guidelines and under consideration of local particularities.

The performance of each QI was expressed as a percentage of adherence to an indicator (median performance of all 10 hospitals, to avoid distorting the results in favour of hospitals with a larger number of patients). An 85% compliance rate was considered as a target value for each QI [15,23,30].

To provide a better overview of the quality of AM prescriptions, we grouped the QIs into three main categories: (i) diagnostics, (ii) therapy and (iii) documentation. For each AM therapy, the performance of the three categories was calculated as a percentage. These percentages represent how many of the applicable QIs in the category were fulfilled: (N_(adherent to all applicable QIs of the category)_/ N _(applicable QIs of the category)_ x 100. The individual performance of AM therapies was then expressed as the median performance of all 10 hospitals for each category.

### Inter-rater reliability

Previously, 12 of the 14 QIs have proven to be suitable in a practice test at German hospitals [29]. As it is important to perform practice tests regarding the suitability of QIs before implementation and use in a new setting, we performed an inter-rater reliability (IRR) test on five sample patient records. These tests should ensure the reproducibility of the data by identifying potential rater bias. Each PPS examiner assessed these five example patients independently, as did three different second examiners (two ID specialists and one senior AMS clinical pharmacist). The percentage of agreement of the answers was expressed as the к coefficient. к coefficients of ≥ 0.61 are regarded as a substantial strength of agreement and к coefficients of ≥ 0.81 as an almost perfect strength of agreement [31]. Quality indicators with к coefficient ≤ 0.6 were excluded from the study.

### Statistics

For descriptive statistics, we used the median frequency of appropriate AM prescribing quality across all 10 hospitals. We calculated the median for each of the 14 QIs as follows: first, we calculated the frequency of appropriate AM prescribing quality at hospital level for each QI. We then used these frequencies to calculate the median frequency across all 10 hospitals. The disadvantage of this approach is that the median frequency cannot be directly translated into a real number of patients, but the advantage is that the median frequency is independent of the size of the hospital and therefore not distorted by the larger hospitals.

In order to detect differences between the individual hospitals in their proportion of fulfilled QIs, a two-sided Pearson's chi-squared test was performed. Since there was a total of 14 tests we used Bonferroni correction to adjust for multiple testing. For these tests, a p value of < 0.00357 (0.05/14) was considered significant after correction. Data analysis was performed using R software version 4.2.0 (R Foundation, Vienna, Austria).

In order to identify potential trends in the quality of AM prescribing throughout 2021, we conducted a descriptive analysis of the differences between the three quarters of the year for each quality indicator, employing a binominal generalised mixed model (GLMM with logit-link). This is a multilevel model with random intercept and two levels using the three quarters, which were the same for every medical centre, as repeated measurements (level 1 variable). The repeated measures are nested within the medical centres, which are the grouping variable (level 2 variable). Two different tests were performed: first, we used a Wald chi^2^ test as an omnibus test to test for global differences between the three quarters. Second, if the global chi-squared tests indicated differences between quarters we used a pairwise comparison with Tukey correction to test for specific differences between two quarters (e.g. Q2 vs Q3). The GLMM were computed using the R package lme4 together with the car package for the Wald test. For pairwise comparisons, the R package emmeans was employed.

## Results

On the survey days, in total 8,560 hospitalised patients were recorded. Among them, 2,861 patients (33%) received at least one AM. The data from these 2,861 patients and the antimicrobials prescribed were further analysed in this study. The overall number of AM prescriptions was 3,500. The median patient age was 72 (interquartile range (IQR): 58—82) years. The cohort was balanced in terms of sex (49% (1,391) female vs 51% (1,467) male). Median length of hospital stay was 13 (IQR: 7—22) days. Further patient characteristics are outlined in Supplementary Table S4.

### Antimicrobial use

**Figure 1:**
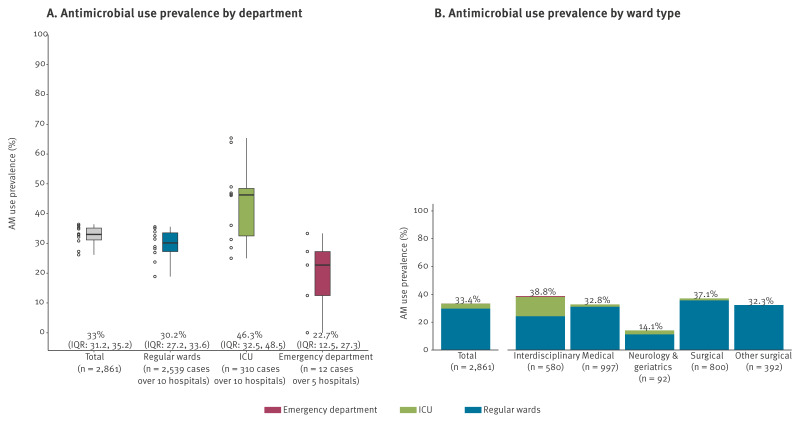
Antimicrobial use prevalence (%) in 10 non-university hospitals by (A) ward type and (B) department, southwest Germany, 2021

ICU: intensive care unit; IQR: interquartile range; n: total number of AM therapies and prophylaxis.

Intensive care units exhibited highest median AM utilisation at 46%, compared with regular wards (30%) and emergency departments (23%) (Figure 1A). For interdisciplinary wards, 39% of patients received AM treatment. The prevalence of AM use was lower in surgical (37%) and medical wards (33%). Neurological and geriatric wards revealed the lowest AM use prevalence with 14% (Figure 1B).

In Figure1A, datapoints represent hospitals, with median of all hospitals and interquartile range shown.

The median duration of therapeutic AM treatments was 7 (IQR: 5–11) days. The vast majority of AM therapies were administered intravenously (median: 82%). Community-acquired infections prevailed, accounting for 55% of all prescriptions. The most frequently treated infections at the 10 participating hospitals were pneumonia (median: 19%) and abdominal infections (median: 15%). Around a fifth of the AM therapies were given in the context of medical (5%) or surgical (13%) prophylaxis. In 23% of AM therapies, combinations of two prescriptions were included, and in 5% more than two were included. Detailed information on AM therapies, indications and underlying infections is provided in Table 2.

**Table 2:** Description of antimicrobial treatments in 10 non-university hospitals, southwest Germany, 2021

Description of AM treatments	Median	IQR
**Total number AM treatments: (n = 3,500)**		
**Duration of AM therapy (days)** (n = 3,500)	7.0	(5.0–11.0)
**Route of application (%)** (n = 3,500)		
Oral	18.5	(13.0–19.2)
Intravenous	81.5	(80.8–87.0)
**Combination therapies^a^ (%)** (n = 3,500)		
Monotherapy	74.9	(48.7–79.5)
Combination therapy with 2 AM	22.7	(17.7–32.2)
Combination therapy > 2 AMs	5.0	(2.0–14.8)
**Indication (%)** (n = 3,499)		
Community-acquired infection	54.8	(46.1–59.6)
Nosocomial-acquired infection	24.0	(21.1–25.1)
Surgical prophylaxis	13.0	(10.6–18.7)
Medical prophylaxis	4.7	(3.6–5.9)
Unknown	2.7	(2.3–5.7)
**Top five infections (%)** (n = 3,497)		
Pneumonia	18.6	(15.5–21.6)
Abdominal infections	14.9	(13.5–18.6)
Skin and soft tissue infections	12.9	(8.3–14.3)
Urinary tract infections	11.2	(9.9–13.4)
Infection not assessable (because of missing information in records)	8.2	(5.2–10.0)

AM: antimicrobial; IQR: interquartile range.

Combination therapies are categorised as monotherapies (therapy with one AM on the day of the survey); combination therapy with 2 AM (therapy with two AMs on the day of the survey); and combination therapy > 2 AM (more than two AM on the day of the survey). Antimicrobials consisting of betalactam/betalactamase inhibitors are categorised as monotherapy.

**Figure 2:**
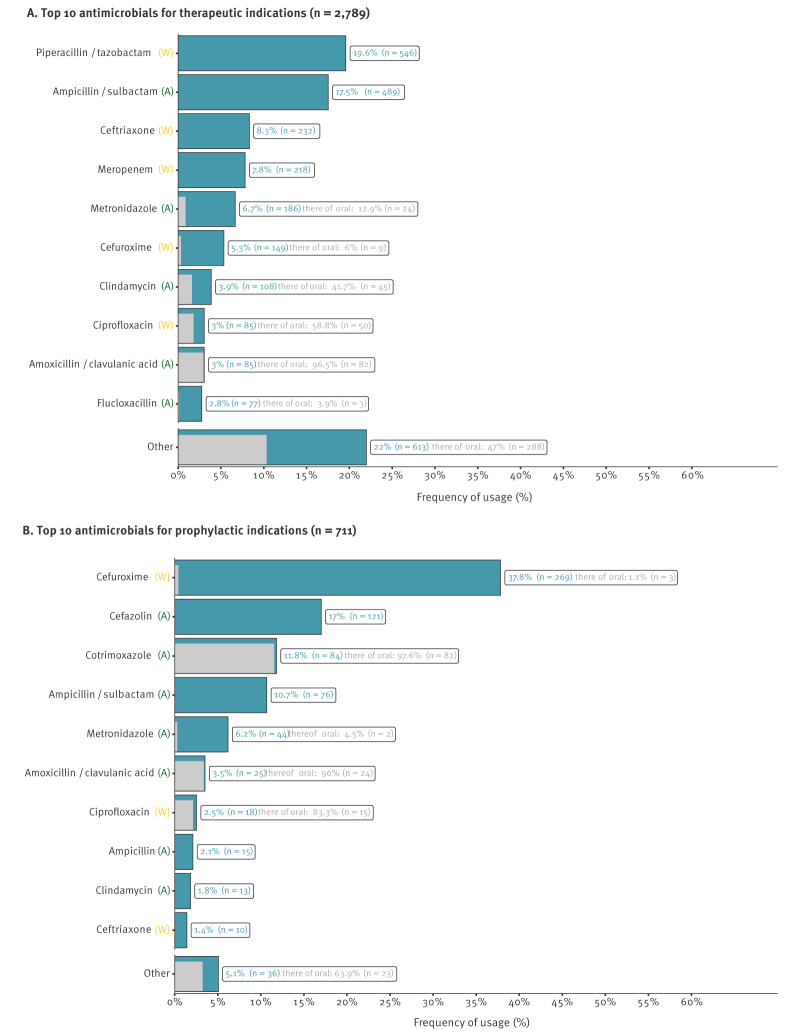
The 10 most frequently prescribed antimicrobials in 10 non-university hospitals (A) for therapeutic indications and (B) for prophylaxis, southwest Germany, 2021

For therapeutic indications (Figure 2A) betalactam antibiotics - specifically piperacillin/tazobactam (20%, 546) and ampicillin/sulbactam (18%, 486) - emerged as the most commonly used agents followed by ceftriaxone (8%, 232) and meropenem (8%, 218). Metronidazole (7%, 186) was the only non-betalactam in the top five prescribed AMs. Figure 2a illustrates the most frequently used AMs for therapeutic indications and delineates the proportion of oral therapy in respective agents.

A: Access (according to the World Health Organization (WHO) AWaRe (Access, Watch, Reserve) classification); W: watch (according to the WHO AWaRe classification) [5].

n = number of therapies/prophylaxis; % = percentage of all therapies/ prophylaxis.

Proportion of parenteral therapies/prophylaxis are indicated as blue bars. Number of parenteral therapies/prophylaxis are indicated as black numbers. Number and proportion of oral therapies/prophylaxis are indicated as light grey.

The colours indicated on the Y axis represent substance class according to the WHO AWaRe classification (green: Access; yellow: Watch.) Note: reserve antimicrobials do not appear in the top 10.

Cefuroxime (38%, 269) and cefazolin (17%, 121) accounted for the majority of the prescriptions in perioperative antibiotic prophylaxis (PAP). Cotrimoxazole was the most frequently used agent for medical antibiotic prophylaxis (12%, 84) (Figure 2B).

The WHO’s AWaRe classification distinguishes between Access, Watch and Reserve antibiotics. For therapeutic indications, Watch antibiotics accounted for 55% (1,532) of the therapies, followed by Access antibiotics with 40% (1,112) (see Supplementary Table S5). This results in an Access-to-Watch ratio of 0.73. Reserve antibiotics accounted for a very small proportion of prescriptions (3%, 73).

### Quality indicator validation

Inter-rater reliability results showed that nine of the selected QIs had an ‘almost perfect’ IRR (IRR: 0.81–1.00). Three QIs had ‘substantial’ IRR (IRR: 0.61–0.80). The reliability of two QIs was assumed based on the literature [15] because those QIs were not applicable to the example cases. The detailed overview on the IRR results is given in Supplementary Table S6.

### Appropriateness of antimicrobial prescriptions

**Figure 3:**
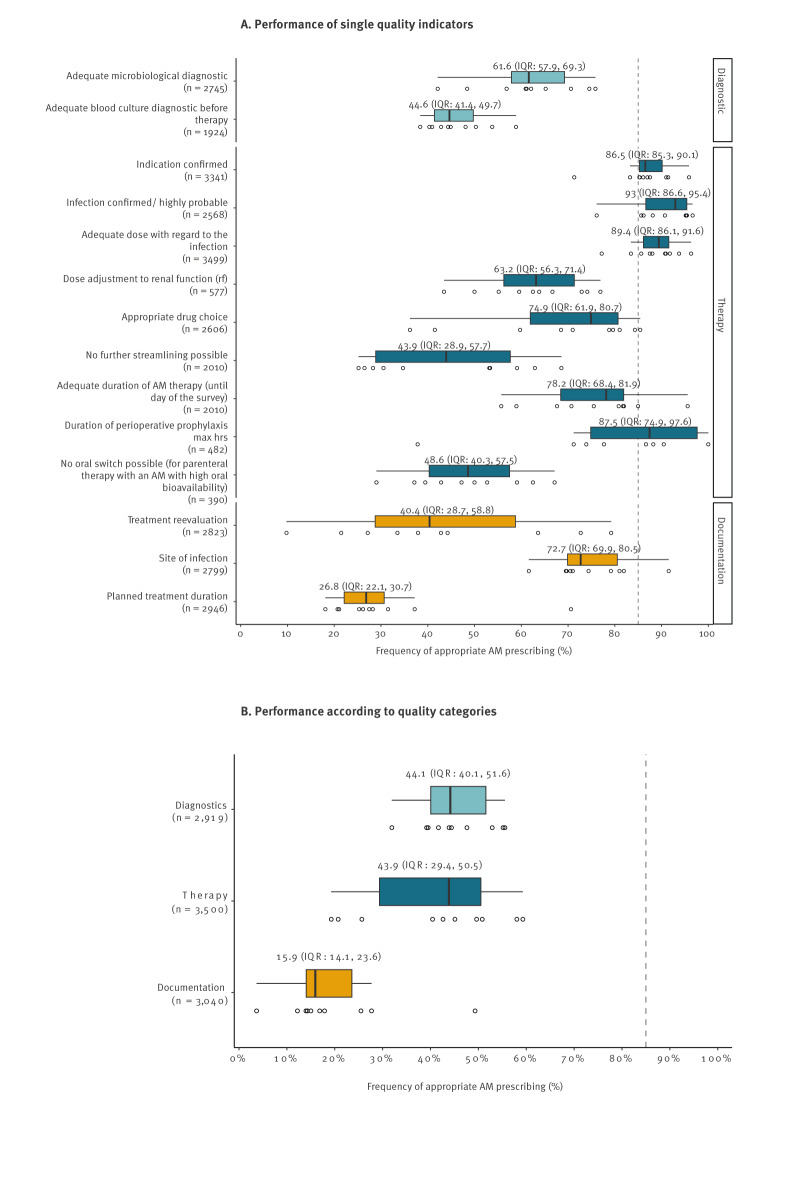
Performance (degree of fulfilment) of quality indicators to assess the appropriate use of AM prescribing in 10 non-university hospitals, for (A) single quality indicators and (B) quality indicator category, southwest Germany, 2021

The different QIs showed considerable range of variation in terms of fulfilment (lowest performance for the QI ‘documentation of planned treatment duration’ (median: 27%) to highest performance for the QI ‘infection confirmed/highly probable’ (median: 93%), Figure 3A). Four of 14 QIs achieved the desired performance level of over 85%. All of them belonged to the therapy category ‘infection confirmed/highly probable’ (median: 93%), ‘adequate dose’ (median: 89%), ‘duration of PAP max 24 hours’ (median: 87%) and ‘indication confirmed’ (median: 86%). The lowest values were found for two QIs in documentation. Reevaluation (median: 40% of patients) and duration (median: 27% of patients) of therapy was documented in the patient medical records within the first 3 days of AM therapy.

AM: antimicrobial.

Performance represents the median performance (%) of the 10 participating hospitals.

Dots represent the results of the 10 individual hospitals; (n cases total = 3,500).

For 3A: n = number of AM therapies that the respective quality indicator was applied to.

For 3B: n = number of AM therapies that could be considered in the respective category.

Other indicators with low adherence were ‘adequate blood culture diagnostic before therapy’ (median: 45%), ‘no further streamlining possible’ (median: 44%) and ‘no oral switch possible’ (median: 49%).

Figure 3B depicts the median fulfilment of the superordinate quality categories. While for the categories of therapy and diagnostics, a median degree of fulfilment of 44% and 44%, respectively, was found, the category documentation had a significantly lower fulfilment level (median: 16%).

### Performance of quality indicators in different hospitals

We detected statistically significant differences between the hospitals in the performance of almost all of the QIs, as well as of the three superordinate categories (all p values < 0.00278 as shown in Supplementary Table S7). Only the performance of two QIs (‘dose adjustment to renal function’ (p = 0.0106) and ‘no oral switch possible’ (p = 0.0094)) showed no significant differences between hospitals after a Bonferroni correction (median: 63% vs 49%).

### Performance of quality indicators over time

The performance of most of the QIs did not change significantly over time during 2021. Supplementary Table S8 gives the results on the calculations. The Supplementary Figure S9 shows the median performance of the QIs for each quarter of 2021. The performance of two QIs showed significant changes over time: ‘adequate microbiological diagnostic’ (p = 0.025) and ‘documentation of planned treatment duration’ (p = 0.007). A pairwise comparison with Tukey adjustment demonstrated a significant difference between Q3 and the Q4 for ‘adequate microbiological diagnostic’. For the QI ‘documentation of planned treatment duration’ we found significant differences between Q2 and Q3 as well as between Q2 and Q4. No discernible trends or patterns emerged across the quarters, suggesting that the observed differences between quarters may not be attributable to a common cause. Therefore, we decided to report our findings only in an aggregated format for the three PPS.

### Appropriateness of antimicrobial prescriptions according to the World Health Organization AWaRe classification

**Figure 4:**
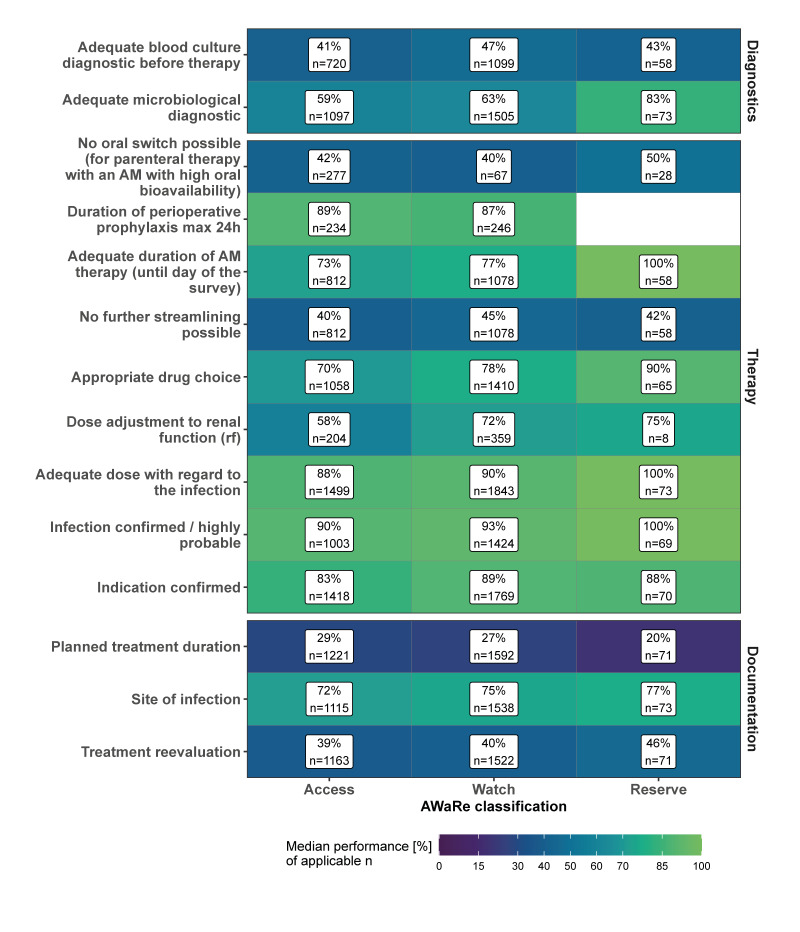
Degree of fulfilment of quality indicators in antimicrobial prescribing in 10 non-university hospitals according to the World Health Organization’s AWaRe classification, southwest Germany, 2021

The rate of the median performance of the different QIs varied between the different drug classes of the WHO’s AWaRe classification (Figure 4). Antimicrobials in the Reserve group showed the highest degree of fulfilment for almost all the QIs (except for ‘documentation of planned treatment duration’ and ‘streamlining’), whereas AMs in the Access group revealed the lowest median performance rate for nine of the 14 tested QIs. The largest differences in the degree of fulfilment were found for the QIs ‘adequate duration of AM therapy’ (Δ Access vs Reserve: 27%), ‘adequate microbiological diagnostic’ (Δ Access vs Reserve: 24%), ‘appropriate drug choice’ (Δ Access vs Reserve: 20%) and ‘dose adjustment to renal function’ (Δ Access vs Reserve: 17%).

AM: antimicrobial; World Health Organization AWaRe classification: Access, Watch, Reserve.

Details are given within the respective white rectangle: n = number of AM therapies that the respective quality indicator was applied to within the respective AWaRe category; Percentages represent median performance (%) of a QI.

Performance represents median performance (%) of the 10 participating hospitals.

## Discussion

This study delivers detailed data on the prevalence and quality of AM use in non-university hospitals in Baden-Württemberg in southwest Germany. Non-university hospitals represent by far the largest part of acute inpatient healthcare in Germany.

Our main findings are as follows: (i) AM use prevalence was relatively high, particularly on regular wards (30%); (ii) most commonly used agents were betalactam/betalactamase inhibitor combinations (40%) for therapeutic indications, and cefuroxime (37%) for AM prophylaxis; (iii) among the WHO’s AWaRe categories, the Reserve group showed the highest QI adherence, while the predominantly used Access and Watch groups showed substantially lower adherence rates; and most importantly, (iv) the quality of AM prescribing requires improvement in most of the aspects studied, especially in the QI categories of ‘documentation’ and ‘diagnostics’.

The median prevalence of AM use in this study was comparatively high at 33%. A repeated PPS in Germany (involving 218 hospitals in 2016 and 250 hospitals in 2022) within the framework of the ECDC PPS found a consistent lower prevalence of 26% [14,32]. The prevalence in our cohort is also higher in a pan-European comparison - the global PPS relating to Western European countries showed a prevalence of 28% [33]. The ECDC PPS reported an overall AM use prevalence of 33% in 2016 and 36% in 2022 in Europe [8,34]. In contrast to the above-mentioned studies, which included different types of hospitals, our study was conducted exclusively in non-university hospitals. Thus, our study adds to the sparse knowledge about AM prevalence in non-university hospitals in Germany [14]. The relatively high AM use in the participating hospitals in our study is a matter of concern and may be due to the fact that there was no AMS team or infectious disease specialist on site at the time the PPS was carried out. In contrast, the above-mentioned study with lower AM prevalence in German hospitals in 2016 notes that at least 28% of the investigated hospitals reported designated staff for AMS [14]. Another influencing factor on the AM use prevalence could be the COVID-19 pandemic, as our study was conducted in 2021. The reported inpatient prescribing of AM in a total of 279 German hospitals increased slightly but significantly during/after the COVID-19 pandemic [35]. This could also be a factor contributing to the relatively high prescription prevalence in our study. However, the seven percentage points higher prevalence in our cohort in comparison with other German hospitals [14,32] can only be partly attributed to this. The proportion of intravenously administered AM was relatively high in our study (81%), but within the range of the most recent reported rates for other German hospitals (83%) [36] and other European countries (79%) [8]. Nevertheless, this is an area with further potential for improvement.

The two substances most frequently used for therapeutic indications were piperacillin/tazobactam (20%) and ampicillin/sulbactam (17%). This AM use pattern was to be expected as these were the most commonly used substances in the latest German ECDC PPS (piperacillin/tazobactam (21%) and ampicillin/sulbactam (13%)) [36] and amoxicillin/betalactamase-inhibitor and piperacillin/tazobactam were the most used agents in the global PPS for Western European countries. However, in Europe amoxicillin/ betalactamase-inhibitor (27%) is used more frequently than piperacillin/tazobactam (11%) [37]. Of note, meropenem was the fourth most frequently prescribed therapeutic AM in the 10 participating hospitals. A further switch from piperacillin/tazobactam to ampicillin/sulbactam, and a reduction of meropenem, should therefore be planned.

The Access-to-Watch ratio for therapeutic indications of the 10 surveyed hospitals was 0.73. Other European high-income countries (Belgium, the Netherlands and the United Kingdom) reported Access-to-Watch ratios higher than 1.0 [37]. Further optimisation of prescribing patterns towards a narrower spectrum is therefore recommended in the studied hospital settings.

Our quality assessment of AM prescriptions showed potential for improvement in most areas. Timely and adequate diagnostics were not performed for the majority of AM therapies.

On time blood culture sampling (if indicated) was performed in only about half of the AM therapies in all the participating hospitals. The degree of fulfilment of this QI varies only slightly between hospitals (IQR: 41–50%). The untimely collection of blood cultures has also been identified as an area for improvement in other studies. Van den Bosch et al. [23] reported that in 22 hospitals in the Netherlands, two blood cultures were taken in a timely manner in only 36% of AM therapies. In a medium-sized hospital in Spain, this quality indicator was fulfilled in 39% of cases [24]. These data suggest lack of knowledge of the blood culture sampling process and its indications [38]. Thus, we recommend planning the implementation of standardised recommendations regarding blood culture sampling.

A treatment plan and the timely documented reevaluation of AM therapy are essential aspects of AMS. Documentation of AM prescriptions was insufficient in our study (sufficient documentation overall: 16%, reevaluation: 40%, planned treatment duration: 27%). Documentation of planned treatment duration was equally inadequate in all participating hospitals (IQR: 22–31%), while the documentation frequency of treatment reevaluation varied widely between hospitals (29–59%). Other recent European studies describe a much higher performance for the indicator ‘documentation of an antibiotic treatment plan’ (22 Dutch hospitals: 61%, nine Belgian/Dutch hospitals: 59%) [23,30]. These targets were achieved even though the Dutch study was also conducted in non-university hospitals, predominantly without electronic patient records and without AMS teams. The need for improvement concerning the documentation in German hospitals has already been described [39]. Especially with respect to AM prescriptions, adequate documentation should be pursued more vigorously. Nevertheless, infection sites were documented in patient records in the majority of cases (73%). Compared with global PPS data and ECDC PPS data, documentation frequency of indications in the hospitals studied is similar to those in other western European countries (80% [34], 83% [8] and other German hospitals in 2022 (74%) [36]).

The right choice of substance, possible streamlining and choosing the recommended route of administration (switching to oral administration in only 49% of possible AM treatments), are also areas for improvement. However, comparable low rates were described in a Spanish study (52%) and even lower rates in a Dutch evaluation (32%) [23,24]. The discontinued implementation of these processes may be attributed to the lack of regular treatment reevaluations. In the recent ECDC PPS, an association between having dedicated staff for AMS (> 0 full-time equivalents (FTE)) and higher presence of a policy for post-prescription review in at least one ward was described [8].

In most cases, there was adequate justification for the use of AM (indication: 86%, infection: 92%). Therefore, these aspects of process quality should not be the main target of future AMS interventions.

Interestingly, PAP was discontinued on time in most cases (87%). Nine of the 10 participating hospitals achieved a degree of fulfilment for this QI of over 70%. The reason for this very positive result in our sample of hospitals is not known. Timely discontinuation of PAP has previously been described as a major quality issue [40] in AM use. In the EU, 48% of PAP are prescribed for more than 1 day [8]. The proportion reported for German hospitals is lower (38%) [36] than in other European countries, but still noticeably higher than in our study (median 13%).

With regard to the WHO’s AWaRe categorisation, differences in the degree of fulfilment of the QIs were identified. With the exception of documentation of the planned duration of therapy, all the QIs were fulfilled more frequently for therapies with substances from the Reserve group, than for those from the Access group. The higher degree of fulfilment may be due to the fact that prescribers act more consciously and prudently when prescribing Reserve substances. Yet, the fact that the duration of therapy for Reserve substances is less frequently specified within the first 3 days should be specifically addressed in AMS intervention reevaluation, including the determination of a treatment plan, and is particularly necessary when using Reserve substances.

Compared with the results of the German ECDC PPS 2022 [36], our findings regarding AM use pattern and the two QIs examined (discontinuation of PAP and documented reason for AM use) are consistent. The prevalence of AM use in our 10 participating hospitals is higher. It should be noted that the German PPS data also include results from seven university hospitals and some hospitals with AMS staffing (median 0% of hospitals with one AM FTE per 250 beds, median number of beds per AM FTE: 1,120). This may be related to the lower prevalence.

As the performance of most QIs varies significantly between the participating hospitals, we recommend that a local quality assessment based on our PPS methodology be carried out before planning the implementation of AMS strategies at other hospitals.

Our study has limitations which need to be considered. The PPS methodology itself has weaknesses. Prolonged patient stays and therapies may include an overrepresentation of more complex patients and therapies. Unforeseen events such as individual prescribing patterns and disease outbreaks may distort results. To minimise this, the survey was conducted three times in each hospital at intervals of 3 to 4 months. There was no external control of the QI assessment (except by the staff of the participating hospitals). However, data collectors were intensively trained in the PPS methodology and the IRR was consistent. The study centres were not randomly selected, but rather the centres were recruited upon an expression of interest to participate in the ID ROLL OUT study [20]. This might have led to a selection bias, but we did include 10 hospitals of different sizes and structures, from different settings, including rural and urban settings, in the federal state of Baden-Württemberg, and with various hospital sponsors/owners. As the lower quality of AM prescribing in non-university hospitals was also shown in an earlier pilot study of AM prescribing quality in German hospitals without AMS teams [15], we hypothesise that our findings are replicable in other non-university hospital settings across Germany. Moreover, this study has several strengths. The multicentric study design and the large sample size (2,861 patients and 3,500 prescriptions) allow a broad insight into AM use with generalisable results. Our thorough and extensive PPS methodology provides further in-depth analysis of AM prescription quality in non-university hospitals in Germany. Rigorous expert plausibility checks contribute to the reliability of the data and the IRR analysis further validates the included QIs. The QI-based approach can identify key areas for prescription improvement across hospitals and can be used to tailor future targeted AMS interventions.

As part of the ID ROLL OUT study, the changes after implementing formal AMS teams in AM prescription quality and use prevalence will be determined in a future PPS. In addition, the effect of the implementing infectious diseases specialist services on AM prescribing will be investigated.

Our PPS methodology might be supported by automated electronic chart reviews in the future. More technical approaches are needed to reduce the time required for the complex PPS. Basic data, such as the number of patients receiving AM, the dose or the route of application, could be collected automatically to allow more time for the sophisticated assessment of QIs by qualified experts.

## Conclusion

This multi-centre PPS in non-university hospitals in southwest Germany showed that there is room for improvement in various aspects of the quality of AM prescribing. Together with the high AM use prevalence in our cohort, our findings emphasise the need for sustained AMS programmes and implementation of infectious diseases specialist services in non-university hospitals in our region – the impact of such interventions will be analysed in the intervention phase of the ID ROLL OUT study. Our detailed PPS approach can identify key targets for future AMS interventions. It should be considered as a valuable tool in the development and validation of AMS programmes.

## Supplementary Data

311000 Supplementary Material
