# Supplementary material for: An in-depth analysis of antimicrobial prescription quality in 10 non-university hospitals, in southwest Germany, 2021
Source: Euro Surveill. 2024 Nov 14;29(46):2400156. doi: 10.2807/1560-7917.ES.2024.29.46.2400156 (PMC11565651; doi:10.2807/1560-7917.ES.2024.29.46.2400156)
Supplement: Supplementary Material [file 24-00156_FOERST_Supplement.pdf]

Supplementary material

This supplementary material is hosted by *Eurosurveillance* as supporting information alongside the article “An in-depth analysis of antimicrobial prescription quality in 10 non-university hospitals, in southwest Germany, 2021”, on behalf of the authors, who remain responsible for the accuracy and appropriateness of the content. The same standards for ethics, copyright, attributions and permissions as for the article apply. Supplements are not edited by *Eurosurveillance* and the journal is not responsible for the maintenance of any links or email addresses provided therein."

Supplementary Table S1: Hospital characteristics

	Number of beds	On-site pharmacy service	On-site microbiology	Prescribing system
Hospital 1	400	No	No	Paper
Hospital 2	401	Yes	No	Paper
Hospital 3	639	Yes	Yes	E-prescribing
Hospital 4	835	Yes	No	Paper
Hospital 5	335	No	No	Paper
Hospital 6	761	Yes	Yes	E-prescribing
Hospital 7	375	Yes	Yes	Paper
Hospital 8	465	No	No	Paper
Hospital 9	282	Yes	No	E-prescribing

Hospital 10	260	No	No	E-prescribing (except ICU)
Beds all hospitals (Median)	400,5			
Large (>600 beds):	30 %			
Medium (350 – 600 beds):	40 %			
Small (<350 beds):	30 %			

Supplementary Table S2: Department's composition

Departments	Specialities
medical	general internal medicine, cardiology, nephrology, pneumology, gastroenterology, haematology, oncology, infectiology, rheumatology
surgical	general surgery, visceral surgery, cardiac surgery, vascular surgery, traumatology, orthopaedics, plastic surgery
neurology and geriatrics	neurology, geriatrics
other surgical	otolaryngology, neurosurgery, urology, ophthalmology, gynaecology
interdisciplinary	wards with < 80 % of patients can be assigned to a certain department

Supplementary Table S3: Quality indicators of the PPS protocol and algorithm

Category<br>Quality indicator	QI applies to	QI does NOT apply to	algorithm

**Diagnostics**			
**adequate blood culture diagnostic before therapy** [1,2,5,7,9]<br><br>per drug<br><br>evaluated on day of the survey	AM therapies for the following infections:<br><br>Pneumonia<br>Bacteremia<br>Bone/joint infections<br>Endocarditis<br>CNS infections<br>Abdominal infections<br>Neutropenic fever<br>Infection of unknown origin	AM therapies for other infections,<br><br>AM prophylaxis	Σ (yes, one or more sets)<br>———<br>Σ (yes, one or more sets+ no)
**adequate microbiological diagnostic** [1,3,4,6,7,9]<br><br>per drug<br><br>evaluated on day of the survey	AM therapies, when microbiological examination is advisable	AM prophylaxis<br><br>AM therapies, when no microbiological examination required	Σ (yes, pathogen identified + yes, result pending or negative)<br>———<br>Σ (yes, pathogen identified + yes, result pending or negative + no, diagnosis incomplete/not performed)
**Therapy**			
**indication confirmed** [1,2]<br><br>per drug<br><br>evaluated on day of the survey	All AM therapies and AM prophylaxis	AM therapies that could not be assessed on the basis of the available data	Σ (yes, confirmed or medically reasonable)<br>———<br>Σ (yes + no)

**infection confirmed/ highly probable** [2,3]<br><br>per drug<br><br>evaluated on day of the survey	All AM therapies	AM Prophylaxis<br><br>AM therapies that could not be assessed on the basis of the available data	Σ (yes confirmed or highly probable)<br>———<br>Σ (yes confirmed or highly probable + no)
**adequate dose with regard to the infection** [4]<br><br>per drug<br><br>evaluated on day of the survey	All AM therapies and AM prophylaxis		Σ (yes, adequate dose)<br>———<br>Σ (yes, adequate dose + no, too high + no too low)
**dose adjustment to renal function (rf)** [1,2,5,6]<br><br>per drug<br><br>evaluated on day of the survey	AM that requires dose adaptation for renal impairment AND patients with renal impairment	AM that do not require adaptation to renal function<br><br>Patients with normal renal function	Σ (yes optimal)<br>———<br>Σ (yes optimal + not optimal + not adapted)
**appropriate drug choice** [1,2,5–7]<br><br>per patient<br><br>evaluated on day of the survey	AM therapies	AM Prophylaxis<br><br>No bacterial or fungal infection	Σ (yes, drug choice concordant with microbiological results or in accordance with guideline)

		AM therapies that could not be assessed on the basis of the available data	Σ (yes, drug choice concordant with microbiological results or in accordance with guideline + no + not assessable because of insufficient diagnostic)
**no further streamlining possible** [1,2,5–7]<br><br>per patient<br>evaluated on day of the survey	AM therapies with known pathogen	AM Prophylaxis<br><br>No bacterial or fungal infection<br><br>AM therapies that cannot be assessed (for any reason)	Σ (no streamlining possible)<br>―<br>Σ (no streamlining possible+ streamlining possible + can be ceased completely)
**adequate duration of AM therapy (until day of the survey)** [1,2,4,6,8]<br><br>(<br>per patient<br>evaluated on day of the survey	AM therapies, that can be assessed	AM Prophylaxis<br><br>No bacterial or fungal infection<br><br>AM therapies that cannot be assessed (for any reason)	Σ (cannot be ceased)<br>―<br>Σ (cannot be ceased+ can be ceased completely)

**duration of perioperative prophylaxis max 24 h** [2–4]<br>per drug<br>evaluated prospective	AM for perioperative prophylaxis	AM therapies<br>AM for medical prophylaxis	$\frac{\Sigma \text{(yes)}}{\Sigma \text{(yes + no)}}$
**no oral switch possible (for parenteral therapy with an AM with high oral bioavailability)** [1,2,5–7,9]<br>per drug<br>evaluated on day of the survey	AM with high bioavailability (Azole-antifungals, ciprofloxacin, clindamycin, cotrimoxazole, doxycycline, levofloxacin, linezolid, metronidazol, moxifloxacin, ofloxacin, rifampicin) when applied iv	AM with low bioavailability<br>AM that are applied orally	$\frac{\Sigma \text{(yes)}}{\Sigma \text{(yes + no)}}$

**Documentation within 72 h**			
**planned treatment duration** [4,5,7]<br><br>per drug<br><br>evaluated retrospective	AM therapies for infections (except neutropenic fever)	AM prophylaxis<br><br>AM therapy for neutropenic fever	Σ (yes)<br>―――<br>Σ (yes + no)
**site of Infection** [4,5,7]<br><br>per drug<br><br>evaluated retrospective	AM therapies	AM prophylaxis	Σ (yes)<br>―――<br>Σ (yes + no)

**treatment re-evaluation**<br>[4,10]<br>per drug<br>evaluated retrospective	AM therapies that can be reevaluated within 72 h	AM prophylaxis<br>AM for neutropenic fever<br>AM for infections that require longer diagnostic than 72 h	$\frac{\Sigma (\text{yes})}{\Sigma (\text{yes + no})}$

Supplementary Table S4: Baseline characteristics of patients included **(Σ all hospitals)**

Patient characteristics	Number	Percentage [%]
in-patients on the survey day	8560	100
Ward type		
regular ward	7843	91.6
intensive care unit	664	7.8
emergency department	53	0.6
Departments[a]		
medical	3040	35.5
surgical	2156	25.2
neurology and geriatrics	654	7.6
other surgical	1215	14.2
interdisciplinary	1495	17.5
**Patients included (in patients with AM use on the survey day)**	**2861**	100
median age [yrs.] (interquartile range)	72 (58-82)	
female	1391	48.7
male	1467	51.3
sex unkown/missing information	3	0.1
median length of stay [days] (interquartile range)	13 (7-22)	

[a]See Table S2 for the department’s composition

Supplementary Table S5: Use of Reserve AM (AWaRe classification of the WHO) [use frequency, pooled data of all participating hospitals]

Antinfective Use	Number total	Percentage [%] total	Number therapeutic	Percentage [%] therapeutic
Total number of AM therapies	3500	100	2789	100
Access	1499	42.8	1112	39.8
Watch	1844	52.7	1532	54.9
Reserve	73	2.1	73	2.6
AM not classified	84	2.4	71	2.5
missing information	1	0	1	0

Supplementary Table S6: Inter-Rater Reliability of QIs

**Category** / **Quality indicator applied**	**IRR**
Diagnostics	
adequate blood culture diagnostic before therapy	0.85
adequate microbiological diagnostic	0.93
Therapy	
indication confirmed	0.95
infection confirmed/ highly probable	0.95
adequate dose with regard to the infection	0.75
dose adjustment to renal function (rf)	0.89
appropriate drug choice	0.81
no further streamlining possible	0.68
adequate duration of AM therapy (until day of the survey)	0.68
duration of perioperative prophylaxis max 24 h	Literature [2] 0.83

no oral switch possible (for parenteral therapy with an AM with high oral bioavailability)	Literature [2] 0.7*
Documentation within 72 h	
planned treatment duration	0.91
site of infection	0.98
treatment re-evaluation	0.89

Supplementary Table S7: differences in the performance of the QIs between the hospitals

performance of the quality indicators [%]; (Minimum, Quantiles, Median, Maximum) of the ten individual hospitals; (N cases total: 3500); n = number of AM therapies, that the respective QI applied; p-value of < 0.00278 indicates significant differences after Bonferroni correction in the performance of the hospitals.

**Category** **Quality indicator applied**	**n**	**Minimum [%]**	**25. Quantil [%]**	**Median [%]**	**75. Quantil [%]**	**Maximum [%]**	**p-value**
Diagnostics							
adequate blood culture diagnostic before therapy	1924	38.40	41.43	44.63	49.74	58.88	< 0.00278
adequate microbiological diagnostic	2745	42.13	57.89	61.62	69.27	75.86	< 0.00278
Therapy							
indication confirmed	3341	71.33	85.28	86.53	90.14	95,87	< 0.00278
infection confirmed/ highly probable	2568	76.13	86.65	92.95	95.40	96.65	< 0.00278
adequate dose with regard to the infection	3499	77.20	86.12	89.41	91.57	96.32	< 0.00278
dose adjustment to renal function (rf)	577	43.48	56.27	63.16	71.35	76.92	0.0106 (n.s.)
appropriate drug choice	2606	36.18	61.94	74.91	80.69	85.44	< 0.00278
no further streamlining possible	2010	25.20	28.86	43.93	57.66	68.58	< 0.00278
adequate duration of AM therapy (until day of the survey)	2010	55.73	68.42	78.17	81.91	95.58	< 0.00278
duration of perioperative prophylaxis max 24 h	482	37.84	74.86	87.45	97.62	100.0	< 0.00278
no oral switch possible (for parenteral therapy with an AM with high oral bioavailability)	390	29.03	40.32	48.61	57.49	67.12	0.0094 (n.s.)

Documentation within 72 h							
planned treatment duration	2946	18.10	22.12	26.82	30.69	70.62	< 0.00278
site of infection	2799	61.62	69.90	72.70	80.55	91.53	< 0.00278
treatment re-evaluation	2823	9.79	28.75	40.37	58.76	79.22	< 0.00278

Supplementary Table S8: differences in the performance of the QIs between the three individual PPS in 2021

Probabilities of appropriate AM prescribing for each quality indicator with Wald χ²-Test results

Quality indicator	Propability of appropriate AM prescribing from GLMM			Type II Wald χ²-Test	
	Q2	Q3	Q4	Wald χ²(2)	*p*
Adequate blood culture diagnostic before therapy	0.484	0.428	0.479	5.05	.080
Adequate microbiological diagnostic	0.635	0.647	0.587	7.38	.025
Indication confirmed	0.865	0.893	0.867	5.01	.082
Infection confirmed	0.922	0.922	0.913	0.74	.690
Duration of perioperative prophylaxis max 24h	0.900	0.844	0.898	3.81	.149
No oral switch possible (for parenteral therapy with an AM with high oral bioavailability)	0.534	0.534	0.400	5.50	.064
Dose adjustment to renal function (rf)	0.647	0.677	0.619	1.33	.515
Adequate dose with regard to the infection	0.896	0.898	0.895	0.07	.967
Appropriate drug choice	0.715	0.727	0.677	4.84	.089
No further streamlining possible	0.441	0.439	0.442	0.01	.995
Adequate duration of AM therapy (until day of the survey)	0.774	0.810	0.754	5.50	.064

Site of infection	0.760	0.744	0.784	3.83	.147
Planned treatment duration	0.260	0.322	0.312	9.99	.007
Treatment reevaluation	0.389	0.442	0.425	4.85	.089

Supplementary Figure S9:

degree of fulfilment of QIs in AM prescribing by time (quarter of the year 2021)

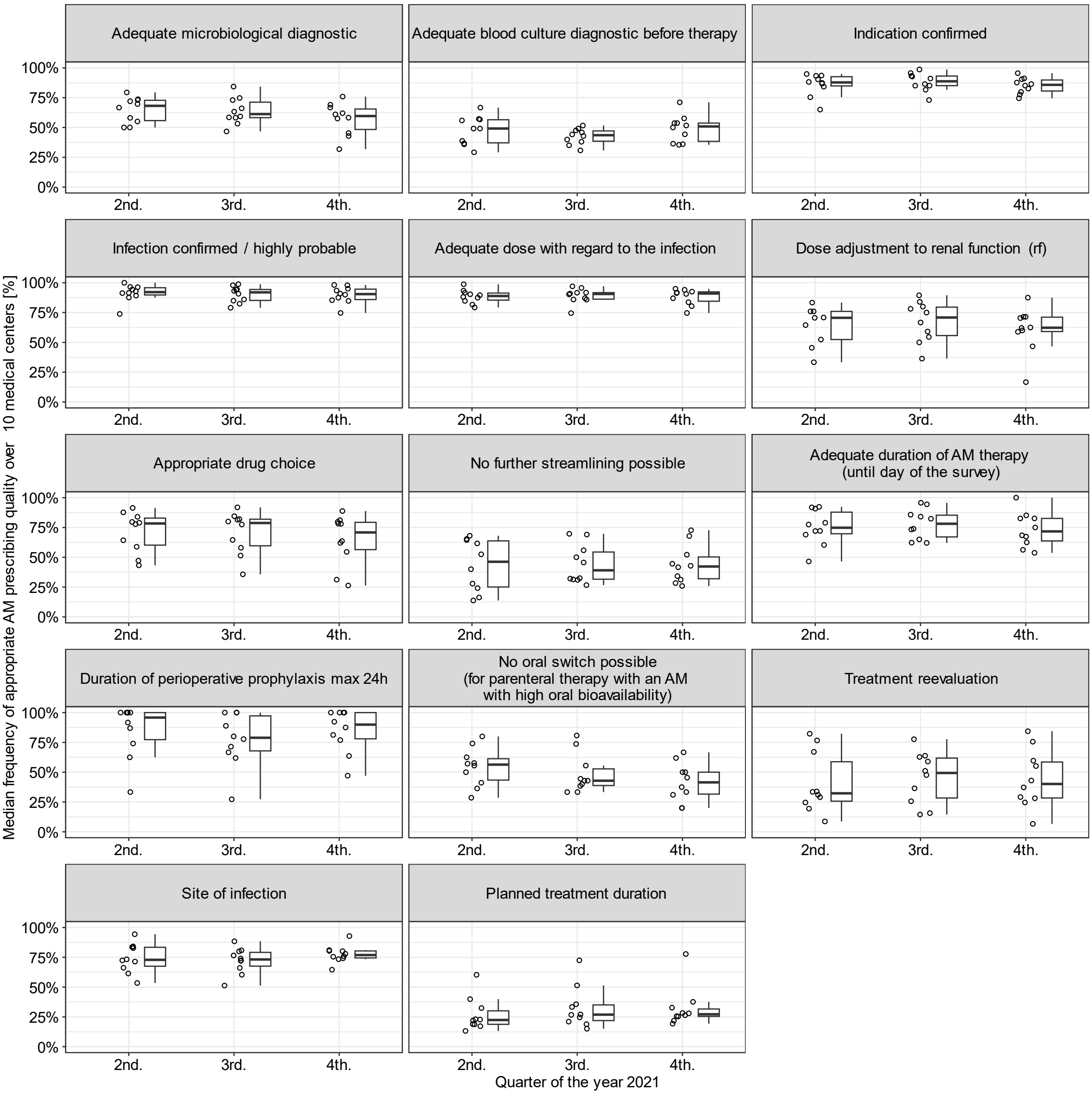

Reference

1. Schouten JA, Hulscher MEJL, Wollersheim H, Braspennning J, B. J. Kullberg, Meer JWM van der, et al. Quality of Antibiotic Use for Lower Respiratory Tract Infections at Hospitals: (How) Can We Measure It? Clin Infect Dis. 2005;41:450–60.

2. Först G, Kern WV, Weber N, Querbach C, Kleideiter J, Knoth H, et al. Clinimetric properties and suitability of selected quality indicators for assessing antibiotic use in hospitalized adults: a multicentre point prevalence study in 24 hospitals in Germany. J Antimicrob Chemother. 2019;74:3596–602.

3. Thern J, De With K, Strauss R, Steib-Bauert M, Weber N, Kern WV. Selection of hospital antimicrobial prescribing quality indicators: a consensus among German antibiotic stewardship (ABS) networkers. Infection. 2014;42:351–62.

4. Monnier AA, Schouten J, Le Maréchal M, Tebano G, Pulcini C, Stanić Benić M, et al. Quality indicators for responsible antibiotic use in the inpatient setting: a systematic review followed by an international multidisciplinary consensus procedure. J Antimicrob Chemother. 2018;73:vi30–9.

5. Arcenillas P, Boix-Palop L, Gómez L, Xercavins M, March P, Martinez L, et al. Assessment of Quality Indicators for Appropriate Antibiotic Use. Antimicrob Agents Chemother. 2018;62:e00875-18.

6. Hermanides HS, Hulscher MEJL, Schouten JA, Prins JM, Geerlings SE. Development of Quality Indicators for the Antibiotic Treatment of Complicated Urinary Tract Infections: A First Step to Measure and Improve Care. Clin Infect Dis. 2008;46:703–11.

7. Van Den Bosch CMA, Hulscher MEJL, Natsch S, Wille J, Prins JM, Geerlings SE. Applicability of generic quality indicators for appropriate antibiotic use in daily hospital practice: a cross-sectional point-prevalence multicenter study. Clin Microbiol Infect. 2016;22:888.e1-888.e9.

8. Van Den Bosch CMA, Geerlings SE, Natsch S, Prins JM, Hulscher MEJL. Quality Indicators to Measure Appropriate Antibiotic Use in Hospitalized Adults. Clin Infect Dis. 2015;60:281–91.

9. Farida H, Rondags A, Gasem MH, Leong K, Adityana A, Van Den Broek PJ, et al. Development of quality indicators to evaluate antibiotic treatment of patients with community-acquired pneumonia in Indonesia. Trop Med Int Health. 2015;20:501–9.

10. Pollack LA, Plachouras D, Sinkowitz-Cochran R, Gruhler H, Monnet DL, Weber JT, et al. A Concise Set of Structure and Process Indicators to Assess and Compare Antimicrobial Stewardship Programs Among EU and US Hospitals: Results From a Multinational Expert Panel. Infect Control Hosp Epidemiol. 2016;37:1201–11.
